# Comprehensive Evaluation of the Salt Tolerance of Soybean Germplasm Resources Cultivated by Heze Academy of Agricultural Sciences

**DOI:** 10.3390/plants15192991

**Published:** 2026-09-30

**Authors:** Wenqian Wang, Yan Liu, Hui Huang, Bangping Liang, Juntao Wang, Baomin Gao, Dajian Zhang

**Affiliations:** 1 Heze Academy of Agricultural Sciences, Heze 274000, China; 2 College of Agriculture and State Key Laboratory of Crop Biology, Shandong Agricultural University, Tai’an 271018, China

**Keywords:** soybean, salt tolerance, agronomic traits, correlation analysis, principal component analysis, membership function

## Abstract

Soil salinization poses a serious threat to global food security, and screening for salt-tolerant soybean germplasm is an effective approach for the efficient utilization of saline–alkali lands. In this study, 116 soybean germplasm accessions developed by Heze Academy of Agricultural Sciences were used as materials. Initial screening for salt tolerance was conducted at the germination stage; then, tolerant accessions were selected and further validated under saline–alkali field conditions at maturity. Comprehensive evaluation was performed via principal component analysis and the membership function method, alongside correlation analysis of agronomic traits. A concentration of 0.5% NaCl (m/v) was determined to be the optimal screening concentration for seed germination, based on which seven strong salt-tolerant materials were selected. Field validation showed that grain weight per plant was strongly correlated with yield under salt stress among the seven accessions tested. The results indicated that Hedou 29 and Hedou 12 exhibited outstanding performance under both evaluation methods. Hedou 29 and Hedou 12 can serve as promising parental materials for salt-tolerant soybean breeding. This study provides empirical data support for breeding salt-tolerant soybean varieties and offers potential cultivar options for soybean production on saline–alkali soils in the Huang-Huai-Hai region.

## 1. Introduction

Soil salinization has become a major abiotic factor restricting global agricultural production. Approximately 1.4 billion hectares of land globally are subject to salinization, with China contributing 10.1% of the world’s total saline–alkali areas [1]. Saline–alkali soils in China are mainly distributed in three major geographical regions: the northwest, the northeast, and the eastern coastal areas [2]. Salt stress causes multiple detrimental effects on plants, primarily manifested as ion toxicity, osmotic stress, and oxidative damage. These effects disrupt cellular functions and interfere with crucial metabolic processes such as seed germination and photosynthesis, ultimately inhibiting plant growth and development and leading to significant yield reduction [3,4]. Therefore, developing and utilizing saline–alkali land resources and breeding salt-tolerant crop varieties have become important approaches to ensuring food security.

Soybean (*Glycine max* (L.) Merr.) is a globally important oil and protein crop, typically classified as moderately tolerant to salinity [5,6]. Despite this tolerance, the crop is not highly resistant; its germination stage is particularly sensitive to salt stress. Exposure to NaCl concentrations between 100 and 200 mmol·L^−1^ has been shown to reduce seed germination by 5% to 23% and markedly inhibit seedling development [7]. At the seedling stage, as salt concentration increases, soybean plants gradually exhibit leaf yellowing, shrinking and curling, and a reduced number of leaves, with plant growth significantly inhibited [8]. Notably, considerable variability in salt tolerance exists across different cultivars and lines, offering a genetic basis for selecting superior germplasm resources suited to saline environments [9,10]. Salt tolerance in soybean is mediated primarily through ion transport regulation and Na^+^/K^+^ homeostasis, as exemplified by *GmCHX1* (ion transporter), *GmSALT3* (restricting shoot Na^+^ accumulation), and *GmCDF1* (negative regulator of salt tolerance at the bud stage) [6,10,11].

Although the Huang-Huai-Hai plain is a major soybean production region in China, the northern part, including the Yellow River Delta and Bohai coastal zones in Shandong (e.g., Binzhou and Dongying), has been increasingly challenged by soil salinization [12,13].

Soil salinization in the Yellow River Delta is dominated by chloride and sulfate–chloride types, with Na^+^ and Cl^−^ as the predominant ions, accounting for 71.03% of total cations and 72.97% of total anions, respectively [14]. In the Yellow River Delta (Dongying City), recent studies have shown that cultivated soils have electrical conductivity of 0.21–12.47 mS/cm and pH of 7.43–9.35, while soil organic matter content is generally low, mostly ranging from 0.4% to 0.8% within the 1-m soil profile [15,16]. Ma et al. found that surface soil salinity peaked in April (mean 1.60 g/kg) and reached its lowest level in October (mean 1.21 g/kg), with strong spring evaporation and summer–autumn rainfall and irrigation identified as the main drivers [17]. Therefore, screening and developing regionally adapted, salt-tolerant soybean varieties is a critical strategy for safeguarding food security and promoting the sustainable utilization of salt-affected land.

Recently, numerous studies evaluating salt tolerance in soybeans have primarily focused on the germination and seedling stages, using common indicators such as germination rate, shoot fresh/dry weight, and plant height [9,18]. To overcome the limitations of single indicators, multi-index comprehensive evaluation methods—such as principal component analysis (PCA), membership function analysis, and cluster analysis—have been increasingly applied to classify salt tolerance levels in soybean germplasm resources [8,19,20]. However, due to differences in the salt tolerance mechanisms of soybean at different growth stages, the correlation between salt tolerance at the germination stage and subsequent field performance may not be significant [21]. For example, Zhang et al. evaluated the salt tolerance of 161 soybean germplasm accessions and found that 35 materials were salt-tolerant at the germination stage, but only eight exhibited high salt tolerance at the seedling stage [22]. Jiang et al. reported that among 798 soybean germplasm accessions, only one showed salt tolerance throughout the entire growth period [23]. These findings indicate that it is difficult to comprehensively assess the salt tolerance potential of germplasm resources based solely on germination stage results. Instead, a combination of field validation and comprehensive analysis of agronomic traits is necessary. Furthermore, systematic studies on the salt tolerance evaluation of major soybean cultivars in specific ecological regions, such as the Huang-Huai-Hai saline–alkali land, remain relatively scarce.

This study used 116 soybean germplasm accessions from the Heze Academy of Agricultural Sciences, which are suitable for planting in the Huang-Huai-Hai region. Firstly, 0.5% (m/v) NaCl was determined as the appropriate salt stress concentration, and the germination-stage salt tolerance of all materials was systematically evaluated. Then, the actual performance of salt-tolerant materials was verified through field experiments. A comprehensive evaluation was conducted by combining agronomic trait correlation analysis, PCA, and the membership function method. The results indicated that grain weight per plant was strongly correlated with final soybean yield in this study, and that Hedou 29 and Hedou 12 showed relatively strong salt tolerance at the germination stage and promising field performance. These findings provide varied support for soybean production in the saline–alkali lands of the Huang-Huai-Hai region and establish a theoretical and material foundation for salt-tolerant soybean breeding.

## 2. Results

### 2.1. Determination of Salt Stress Concentration for Soybean Germination Stage

**Figure 1:**
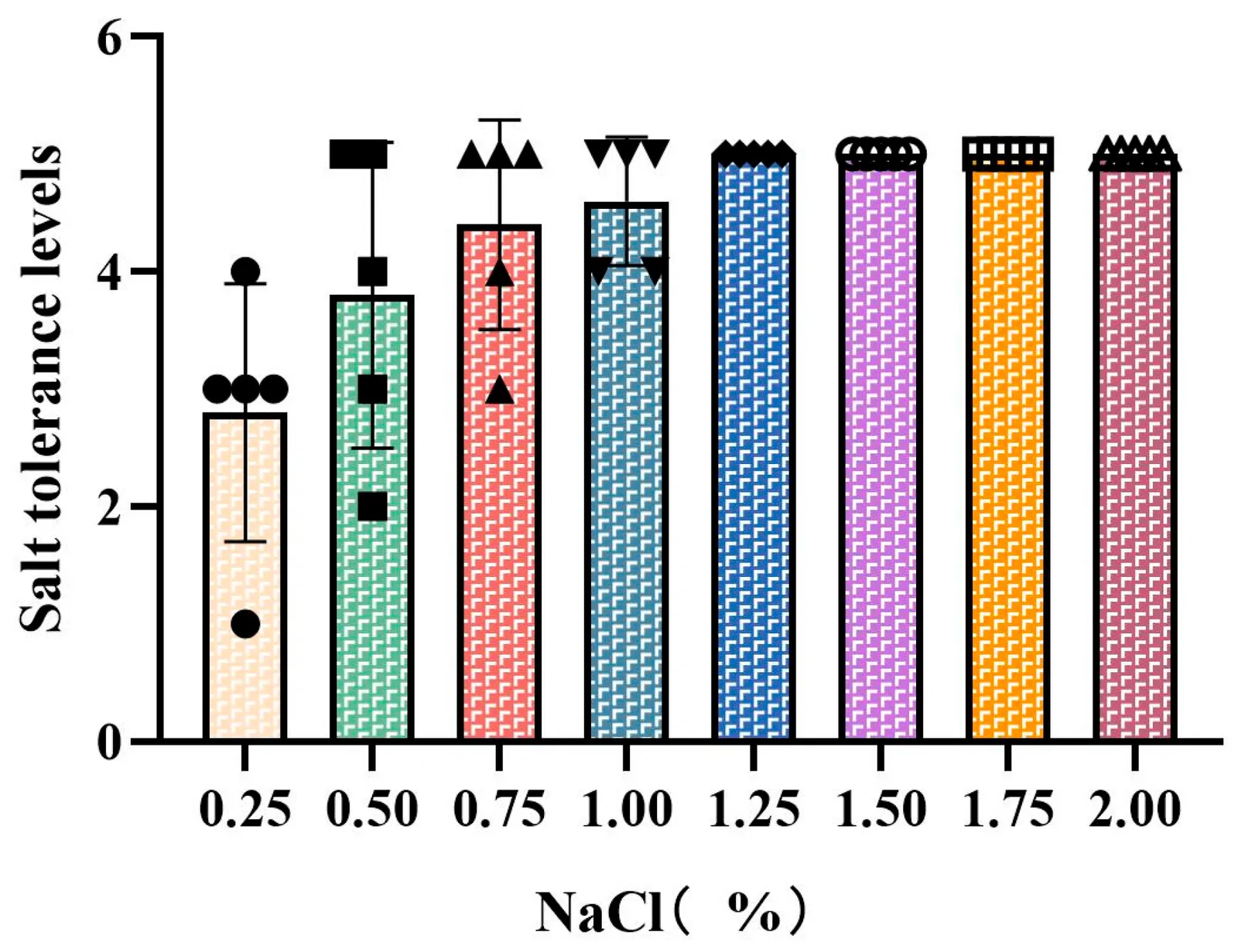
The salt tolerance levels of five soybean cultivars under different NaCl concentrations. Note: The five cultivars were Hedou 12, Hedou 33, Hedou 37, Hedou 38, and Hedou 45. The different colors in the bar chart represent different NaCl concentrations. Data were presented as means ± standard deviation (*n* = 5).

To determine the optimal salt stress concentration, five soybean cultivars, namely Hedou 12, Hedou 33, Hedou 37, Hedou 38, and Hedou 45, were randomly selected. Seed germination experiments were conducted at different NaCl concentrations of 0.25%, 0.50%, 0.75%, 1.00%, 1.25%, 1.50%, 1.75%, and 2.00% (m/v), with water treatment serving as the blank control. The relative germination rate was calculated and converted into salt tolerance levels (Figure 1). The results indicated that after the NaCl concentration reached 1.25% (m/v), the salt tolerance levels of all five cultivars were grade 5. This indicated that the tested cultivars were extremely sensitive to salt stress at this concentration, with their seed germination severely inhibited, which made it impossible to accurately evaluate the salt tolerance level of the experimental materials. Therefore, this concentration and higher concentrations were not suitable for subsequent screening. When the NaCl concentration was 0.25%, 0.50%, 0.75%, or 1.00% (m/v), the average salt tolerance levels of the five soybean cultivars were 2.8, 3.8, 4.4, and 4.6, respectively, with standard deviations of 1.10, 1.30, 0.89, and 0.54, respectively. Among these concentrations, the maximum standard deviation was observed at 0.50% (m/v) NaCl, reflecting that the dispersion of salt tolerance levels of different cultivars was the highest at this concentration. Selecting this concentration as the test concentration for subsequent salt stress experiments can maximize the differentiation of salt tolerance characteristics among different soybean germplasm accessions.

### 2.2. Assessment of Salt Tolerance for Soybean Germplasm at Germination Stage

**Figure 2:**
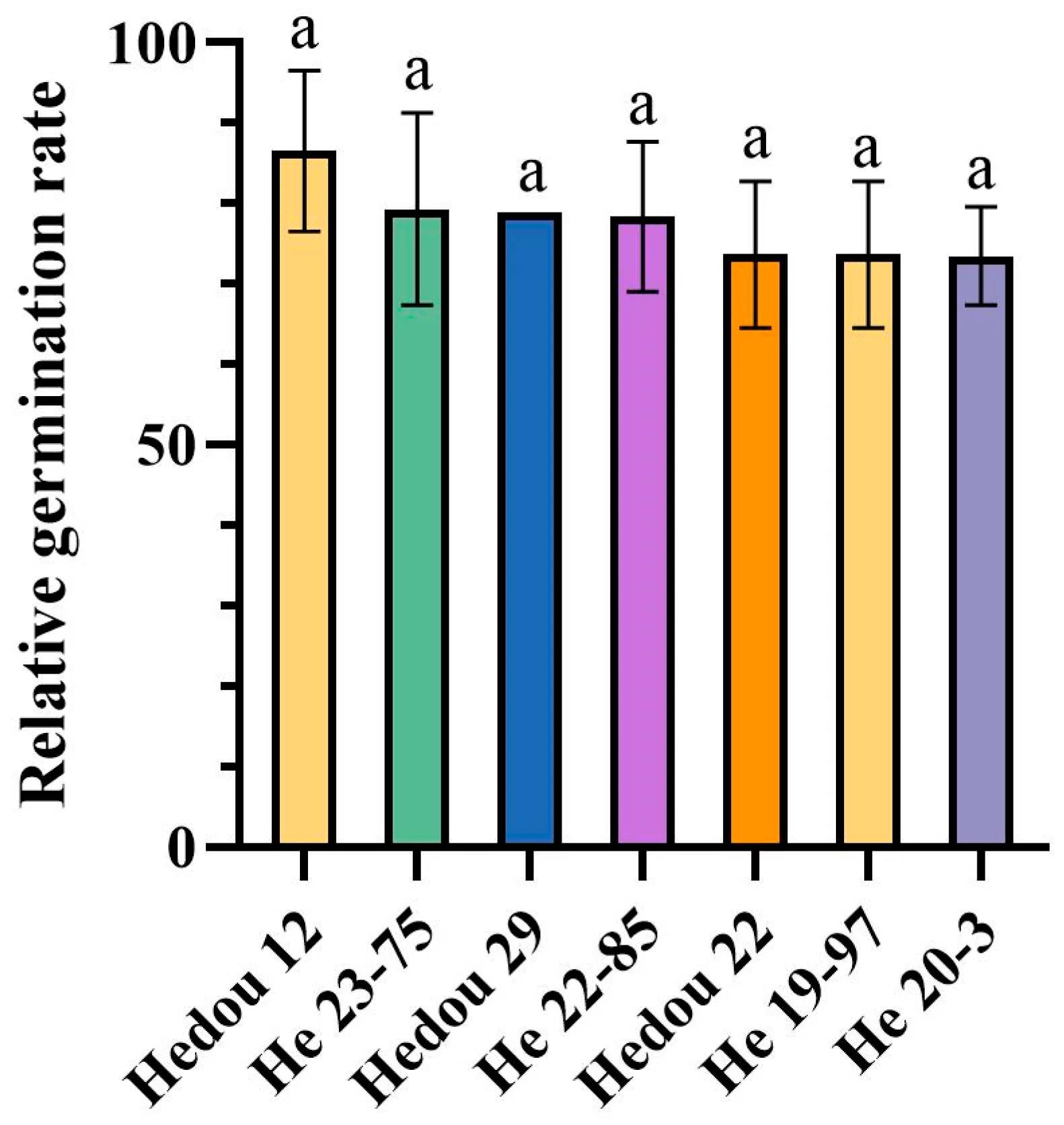
The relative germination rate of the seven strongly salt-tolerant soybean germplasm accessions. Note: Different colors represent different germplasms. Different letters above the error bars indicate a significant difference between means, while the same letter indicates no significant difference (One-way ANOVA with Duncan’s test, *p* < 0.05). Data were presented as means ± standard deviation (*n* = 3).

All soybean experimental materials were subjected to salt stress using 0.50% (m/v) NaCl solution, with the group germinated in sterile water serving as the control. The salt tolerance level was determined based on the relative germination rate, and the salt tolerance type was subsequently classified. As shown in Table S1, among the 116 tested germplasm accessions, seven exhibited strong salt tolerance, 27 displayed moderate salt tolerance, 51 were classified as salt-sensitive, and 31 as extremely salt-sensitive. According to Figure 2, among the seven soybean germplasm accessions with strong salt tolerance, Hedou 12 exhibited the highest level of salt tolerance, achieving a relative germination rate of 86.54%. The other accessions, ranked by their relative germination rates from high to low, were He 23-75, Hedou 29, He 22-85, Hedou 22, He 19-97, and He 20-3.

### 2.3. The Field Performance of Salt-Tolerant Soybeans

**Figure 3:**
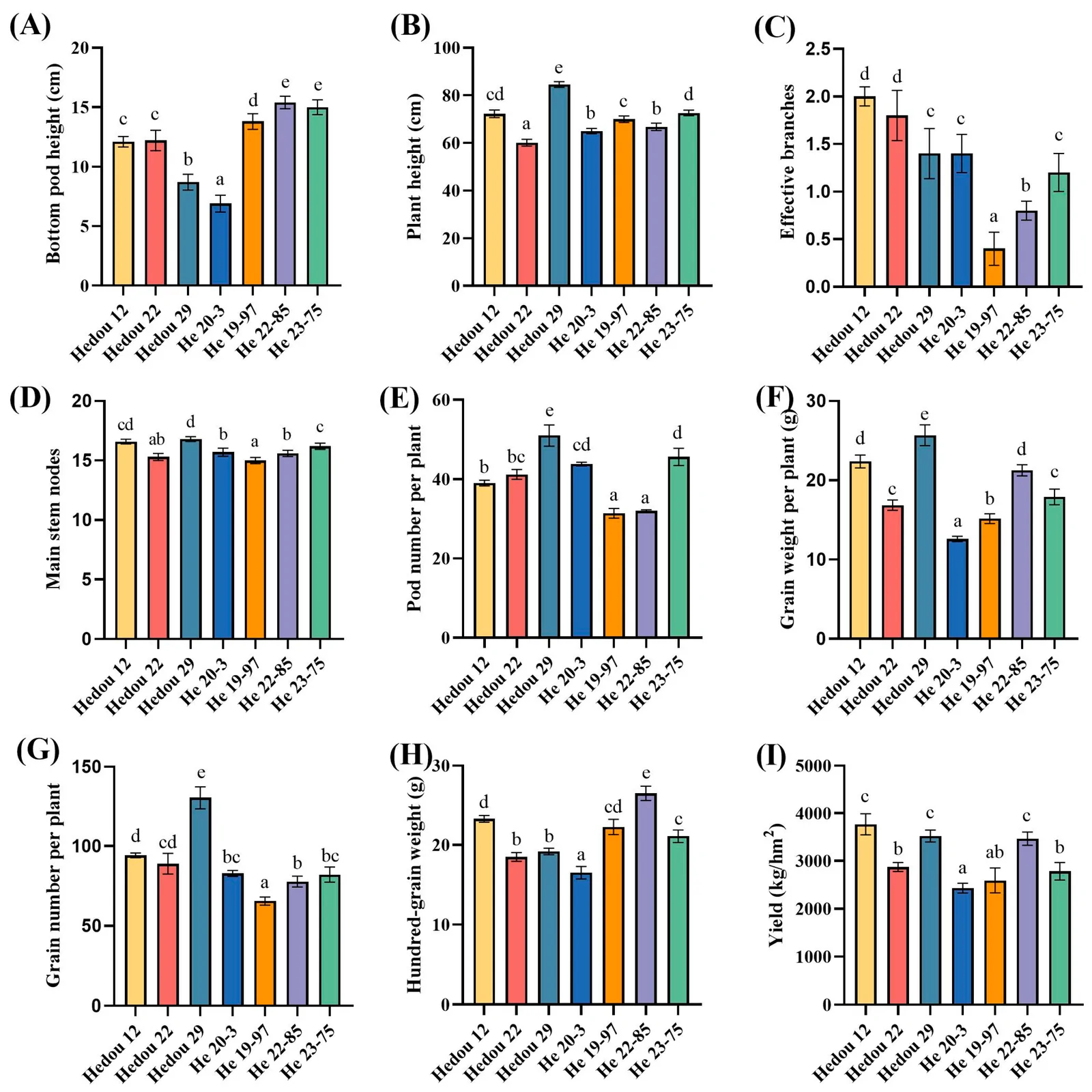
The agronomic traits of seven soybean germplasm accessions. Note: (**A**–**I**) respectively represent the bottom pod height, plant height, effective branches, main stem nodes, pod number per plant, grain weight per plant, grain number per plant, hundred-grain weight, and yield. Different colors represent different germplasms. Different letters above the error bars indicate a significant difference between means, while the same letter indicates no significant difference (One-way ANOVA with Duncan’s test, *p* < 0.05).Data were presented as means ± standard deviation (*n* = 3).

The above-mentioned seven soybean germplasm accessions—Hedou 12, Hedou 22, Hedou 29, He 20-3, He 19-97, He 22-85, and He 23-75—were ultimately selected for field planting trials in the saline–alkali experimental area of Dongying. Among them, only Hedou 29 and He 20-3 had a bottom pod height of less than 10 cm, whereas the bottom pod heights of the remaining germplasm accessions were all above 10 cm (Figure 3A). Other agronomic traits also exhibited considerable variation: plant height ranged from 60.1 to 84.6 cm (Figure 3B), effective branches from 0.4 to 2.0 (Figure 3C), main stem nodes from 15.0 to 16.8 (Figure 3D), pods per plant from 31.4 to 51.0 (Figure 3E), grain weight per plant from 12.59 to 25.67 g (Figure 3F), grain per plant from 65.5 to 130.5 (Figure 3G), hundred-grain weight from 16.50 to 26.52 g (Figure 3H), and yield from 2429.50 to 3767.55 kg/hm^2^ (Figure 3I). Considering the significant differences observed among soybean germplasm accessions in various agronomic traits, further in-depth investigation is still required regarding the intrinsic correlations between these traits, as well as how to construct a comprehensive evaluation system for determining the quality of salt-tolerant soybean germplasm accessions in saline–alkali land.

### 2.4. Correlation Analysis of Soybean Agronomic Traits

**Figure 4:**
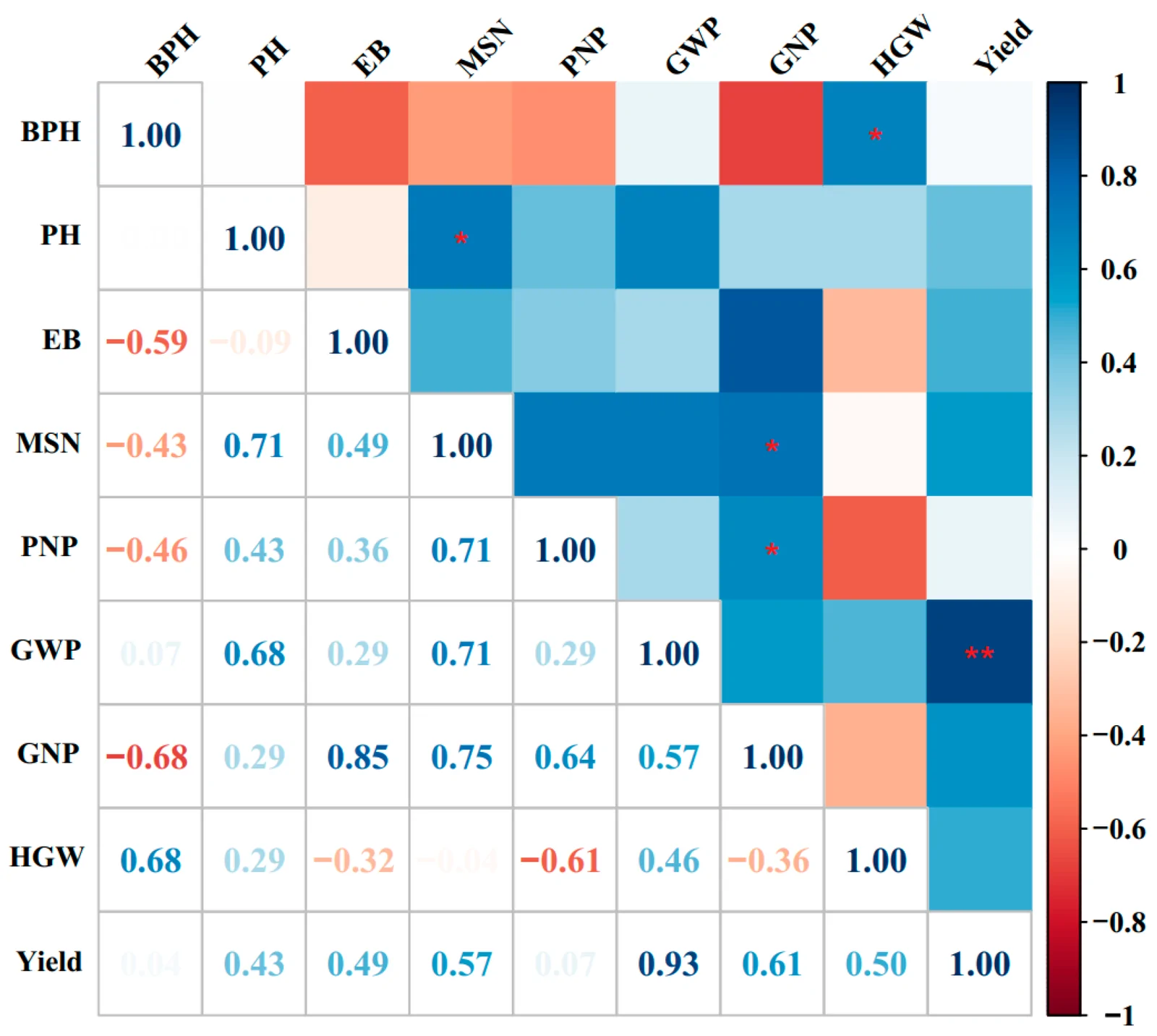
Correlation analysis of 9 agronomic traits of 7 soybean germplasm accessions. Note: BPH, bottom pod height. PH, plant height. EB, effective branches. MSN, main stem nodes. PNP, pod number per plant. GWP, grain weight per plant. GNP, grain number per plant. HGW, hundred-grain weight. * Significantly correlated at the 0.05 level. ** Significantly correlated at the 0.01 level. The shade of color and the number represent the correlation coefficient.

Spearman correlation analysis was used to analyze the correlations among nine agronomic traits of soybeans to clarify the intrinsic connections among them. The results are shown in Figure 4. The correlation analysis results indicated that several agronomic traits of the tested materials exhibited significant positive correlations. Specifically, plant height was significantly positively correlated with main stem nodes, bottom pod height with hundred-grain weight, and grain number per plant with main stem nodes as well as pod number per plant (*p* < 0.05). These findings suggest that directional selection for a single trait can facilitate the coordinated improvement of multiple agronomic traits in soybean breeding. Furthermore, grain weight per plant showed an extremely significant positive correlation with yield (*p* < 0.01), and the correlation coefficient between the two was the highest, reaching 0.93. The above results fully demonstrate that grain weight per plant is a key agronomic trait directly affecting the final yield of soybean, and also provide experimental data support for focusing on and improving this trait in future high-yield soybean breeding.

### 2.5. Comprehensive Evaluation of Salt Tolerance of Soybean Germplasm Accessions Based on Principal Component Analysis

PCA was conducted on nine agronomic traits of soybean, and the results were shown in Table 1. The cumulative contribution rate of the first two principal components reached 81.20%, indicating that these two principal components could cover 81.20% of the information of the original nine traits, effectively achieving data dimensionality reduction. Among them, the eigenvalue of the first principal component is 4.5444, with a contribution rate of 50.49%. Its main determining traits were grain number per plant (eigenvector = 0.45), main stem nodes (eigenvector = 0.4367), and pod number per plant (eigenvector = 0.3682), comprehensively reflecting the podding capacity of the plants. Therefore, it was called the podding-related factor. The eigenvalue of the second principal component is 2.7632, with a contribution rate of 30.7%. Its main determining traits were hundred-grain weight (eigenvector = −0.5783), yield (eigenvector = −0.4196), and grain weight per plant (eigenvector = −0.3773), which mainly reflect grain filling and yield components. Hence, it was defined as the yield-related factor. Based on the above analysis, the nine original traits were converted into two independent comprehensive indicators, and the principal component scores PC1 and PC2, as well as the comprehensive score PC, were calculated. The results are shown in Table 2. The higher the comprehensive score PC is, the better the overall salt tolerance performance of the variety is. The comprehensive scores of the tested germplasm accessions from high to low are as follows: Hedou 29, He 20-3, Hedou 12, Hedou 22, He 23-75, He 22-85, and He 19-97.

### 2.6. Comprehensive Evaluation of Salt Tolerance of Soybean Germplasm Accessions Based on Membership Function Analysis

The fuzzy membership function method was adopted to comprehensively evaluate the salt tolerance of the tested soybean materials. Firstly, the membership function values of each measured trait were calculated for each material, and after eliminating the effects of different dimensions, the values were summed to obtain the comprehensive membership value. A higher comprehensive membership value indicates stronger salt tolerance of the materials. Based on the size of the comprehensive membership value, the ranking results of the salt tolerance of the tested materials were shown in Table 3, from strong to weak, as follows: Hedou 29, Hedou 12, He 23-75, He 22-85, Hedou 22, He 20-3, and He 19-97.

## 3. Discussion

The saline soils in the eastern coastal region of Shandong Province are of the chloride type, due to marine influence, with Na^+^ and Cl^−^ as the dominant ions, and NaCl accounting for approximately 46.69% of the total content [14,24]. Therefore, based on these regional characteristics, this study selected NaCl as the representative salt for salinity stress treatment. In terms of determining the concentration for salt tolerance testing, previous studies usually only set up one or at most four concentration gradients [8,18,19,20]. In contrast, this study used eight different NaCl treatment concentrations, aiming to more precisely screen the appropriate treatment concentration, thereby maximizing the ability to distinguish the salt tolerance differences among different soybean varieties. By statistically analyzing the relative germination rates of different soybean varieties under various salt concentrations and converting these into salt tolerance levels, we found that the dispersion of salt tolerance levels among varieties was highest at a sodium chloride concentration of 0.50% (m/v) (Figure 1). Therefore, 0.5% (m/v) NaCl was determined to be the optimal concentration for large-scale screening of soybean salt tolerance. This concentration is consistent with that used by Zhang et al. in their screening of salt tolerance in soybean germplasm resources in the Huang-Huai-Hai region [22], indicating that the selected concentration in this study is scientifically reasonable.

Through salt tolerance screening at the soybean seed germination stage, a total of seven soybean germplasm accessions with high salt tolerance were obtained, with their relative germination rates ranging from 73.47% to 86.54%, among which Hedou 12 exhibits the highest relative germination rate (Figure 2). However, Xu et al. found this variety to be salt-intolerant when converting the seedling-stage stress index into tolerance levels [25], indicating inconsistent assessment results between the germination and seedling stages. This inconsistency may arise from the limitations of using a single growth stage or a single indicator. Salt tolerance is a complex trait regulated by multiple genes [11], making it difficult to comprehensively evaluate germplasm based on one or two indicators alone. Therefore, a comprehensive evaluation should be conducted based on multiple agronomic traits in the field. However, most current studies focus on a single stage (mainly germination or seedling) or employ a single identification method, whereas comprehensive evaluations spanning from germination to maturity remain rare. Based on the screening results during the germination stage, seven salt-tolerant varieties were planted in the saline–alkali soil in Dongying. Among the seven accessions tested in this study, Hedou 12 had the highest yield, which was 3767.55 kg/hm^2^ (Figure 3I). The yield of Hedou 12 in Heze, Shandong Province, was 4140 kg/hm^2^ [26]. Compared with its yield in the saline–alkaline field in Dongying, the yield decreased by 9.00%, suggesting that the salinity level had a relatively small effect on the yield of Hedou 12. Qihuang 34, a cultivar recommended for saline–alkaline soils, has been reported to yield 4539 kg/hm^2^ in Kenli District, Dongying [27]. This suggests that Hedou 12, although selected as a cultivar suitable for mildly to moderately saline–alkaline soils [22], still performs below salt-tolerant cultivars under actual saline–alkaline field conditions. However, the planting area in this study was relatively small, and the performance of Hedou 12 in saline–alkaline soils needs to be further validated over larger areas. In addition to Hedou 12, the higher-yielding accessions were Hedou 29 and He 22-85 (Figure 3I). Notably, the maternal parent of Hedou 29 is Hedou 12, and the maternal parent of He 22-85 is Qihuang 34, suggesting that using salt-tolerant or high-yielding cultivars as maternal parents is an effective approach for improving salt tolerance and yield in soybean. Analysis of yield-related traits among the three accessions showed that Hedou 29 had the highest pod number per plant, grain weight per plant, and grain number per plant, but the lowest hundred-grain weight (Figure 3), suggesting that its grains were relatively small and that salinity may have had a greater effect on its grain development. In addition, Hedou 29 had a lower bottom pod height, which may partly explain why its yield was not the highest.

The agronomic traits at the maturity stage were analyzed to determine their correlations. The results showed that the single plant grain weight per plant was significantly positively correlated with yield (*p* < 0.01). This finding is consistent with the research conducted by Li on 12 salt-tolerant soybean varieties widely cultivated in Tianjin, which also reported a highly significant positive correlation between yield and plant grain weight [28]. This indicates that a greater plant grain weight reflects a more abundant dry matter accumulation, providing a solid foundation for yield increase. Therefore, this trait might be worth considering as a candidate indicator for high-yield potential in field sampling and selective breeding, although the small sample size (*n* = 7) limits the strength of this conclusion and further validation with larger populations is needed. A limitation of this study is that soil electrical conductivity was not measured, and the salinity level of the experimental field was characterized only by the water-soluble salt content. The measured water-soluble salt content 1.53 g/kg fell within the range reported for this region [17], suggesting that the soil electrical conductivity may also lie within a similar range. However, this inference should be interpreted with caution, and future studies should include direct electrical conductivity measurements to more accurately quantify the salinity level.

PCA effectively avoids the limitation of single-indicator evaluation, whereas the membership function method removes interference from scale disparities among indicators via normalization [24,29]. Combining the two methods enabled an objective comparison and comprehensive ranking of salt tolerance among the tested soybean materials. The ranking of the seven accessions based on germination-stage salt tolerance (Figure 2) did not fully match their field performance at maturity (Table 2 and Table 3). Salt tolerance in soybean is developmental-stage-specific, and the link between early-stage and later-stage tolerance is often weak [21]. Our results support this. Germination-stage screening alone cannot reliably predict field performance; whole-growth-period evaluation under field conditions is more useful for selecting salt-tolerant cultivars. Based on the evaluation results derived from integrating both methods, Hedou 29 and Hedou 12 ranked in the top positions (Table 2 and Table 3), indicating their relatively strong salt tolerance. These two varieties showed promising performance under the saline–alkali conditions of this study and may have potential for cultivation in similar saline–alkali environments, and could serve as potential parental lines for salt-tolerance breeding programs. However, these conclusions are preliminary, as the field validation was conducted at only one saline–alkali location in a single year and involved only seven accessions selected from the germination-stage screening; further multi-location, multi-year trials are needed before broader recommendations can be made. However, it is worth noting that the bottom pod height of Hedou 29 is less than 10 cm (Figure 3A). This trait may limit the efficiency of mechanical harvesting, thereby increasing the risk of yield loss. This also suggests that, when selecting parents for subsequent hybrid breeding, those with higher bottom pod height should be prioritized for hybridization with Hedou 29 to improve its traits. Although He 19-97 ranked lowest in both evaluation methods (Table 2 and Table 3), determining whether its salt tolerance is genetically stable is difficult using only one year of field data, since environmental factors—such as location, soil, and climate—can all influence phenotypic expression. Subsequent field evaluations for salt-tolerant soybean germplasm are planned in diverse saline–alkaline environments, aiming to identify elite accessions suitable for hybrid breeding. In parallel, the integration of molecular marker-assisted selection will expedite the breeding process for salt tolerance. Collectively, the findings are expected to furnish a scientific rationale for optimizing varietal deployment in salinized agricultural lands.

## 4. Materials and Methods

### 4.1. Plant Materials

Twenty-one soybean varieties and 95 lines that were cultivated and stored by the Heze Academy of Agricultural Sciences were utilized to assess salt tolerance. The details of the germplasm accessions were listed in Table S2.

### 4.2. The Identification of Salt Tolerance at the Germination Stage

Five soybean cultivars (Hedou 12, Hedou 33, Hedou 37, Hedou 38, Hedou 45) were selected to determine the optimal salt stress concentration by examining their salt tolerance index under different salt concentrations. The specific steps were as follows: firstly, the soybean seeds were surface-sterilized with 1% NaClO (w/v) for 2 min, followed by washing six times with sterilized distilled water. To confirm the successful surface sterilization, an aliquot of 100 µL of water from the final rinse was plated on LB plates, incubated at 28 °C for 72 h, and observed for the presence or absence of microbial colonies [30].

After surface sterilization, twenty surface-undamaged soybean seeds were placed on filter paper in a Petri dish (10 cm × 10 cm) containing 20 mL of NaCl solution, with concentrations set at 0.25%, 0.50%, 0.75%, 1.00%, 1.25%, 1.50%, 1.75%, and 2.00% (m/v). The seeds were germinated in the dark at 28 °C for 72 h, with three replicates per treatment. The control group used sterile distilled water instead of NaCl solution. Germination was defined as radicle length exceeding half the seed length [31]. The calculation of the relative germination rate of soybeans using Equation (1) indicates the salt tolerance of soybeans:

Relative germination rate (%) = number of germinated soybeans in the treatment group/number of germinated soybeans in the control group × 100.(1)

According to the relative germination rate, the reference standard for the salt-tolerant level of soybean at the germination stage was classified as shown in Table 4. For the screening of large-scale salt-tolerant germplasm resources, the experimental procedures were the same as above, and the concentration of NaCl was set at 0.50% (m/v).

### 4.3. The Identification of Salt Tolerance in the Field Experiment

Field experiments were conducted in Kenli District, Dongying City, Shandong Province (116°24′ E, 39°55′ N) from 20 June to 27 October 2025. After wheat harvest, straw was returned to the field using a straw returning machine (Hebei Gengyun Agricultural Machinery Manufacturing Co., Ltd., Xingtai, China), and a compound fertilizer (N:P_2_O_5_:K_2_O = 15:15:15) was broadcast at 600 kg/hectare as basal fertilizer. The field was then rotary-tilled twice, followed by sowing with a plot seeder. Irrigation was applied using micro-sprinkler tape. At harvest, the soil pH was 8.40 with 44.4 mg/kg hydrolyzable nitrogen, 62.0 mg/kg available phosphorus, 157 mg/kg available potassium, 9.25 g/kg organic matter, 0.528 g/kg total nitrogen, and 1.53 g/kg total water-soluble salt content. The experiment was conducted using a completely randomized block design with three replications. Each plot covered an area of 15 m^2^, arranged into 6 rows with a row length of 5 m and row spacing of 0.5 m, corresponding to a planting density of 240,000 plants per hectare. For agronomic trait investigation, 10 consecutive plants with normal growth and no missing plants were selected from one of the middle four rows of each plot. For bottom pod height, plant height, effective branches, main stem nodes, pod number per plant, grain weight per plant, and grain number per plant, the 10 plants were treated as subsamples within a plot, and their values were averaged to obtain one plot-level value. For hundred-grain weight and yield, one value was directly measured for each plot. The plot was used as the experimental unit. Three plots (replications) were used per treatment, and the three plot-level values were treated as biological replicates (*n* = 3) for statistical analysis and figure preparation. For each replication of each cultivar (line), the middle 4 rows of the plot were harvested and threshed to determine plot yield, with a yield-measuring area of 10 m^2^. The grain weight obtained from indoor agronomic trait investigation was included in the total yield. Finally, the plot yield was converted to grain yield per hectare (kg/hm^2^).

### 4.4. Data Analysis

Basic analysis of the experimental data was performed using Microsoft Excel software (v16.0), while bar charts were generated with Prism software (v8.0.2). Correlation analysis and PCA were performed using RStudio software (v2026.08), based on the plot-level mean values of the seven accessions (Hedou 29, Hedou 12, He 23-75, He 22-85, Hedou 22, He 20-3, and He 19-97; *n* = 7). The input traits were bottom pod height, plant height, effective branches, main stem nodes, pod number per plant, grain weight per plant, grain number per plant, hundred-grain weight, and yield. Membership function analysis was carried out in Excel using the same dataset, with the membership function value using Equation (2):

X(μ) = (X − X_min_)/(X_max_ − X_min_) (2)

In Equation (2), X is the measured value of the index, and X_min_ and X_max_ are the minimum and maximum values of that indicator across all tested materials. Finally, the membership values for each indicator of every material were summed to obtain the total.

## Figures and Tables

**Table 1:** Principal component matrix, eigenvalues, and contribution rates of nine agronomic traits in soybean.

Indicator	PC1	PC2	PC3	PC4	PC5	PC6	PC7
BPH	−0.2237	−0.439	0.0487	−0.7272	0.3171	0.1815	−0.00441
PH	0.3518	−0.1613	−0.575	−0.0331	−0.1918	0.6531	0.1138
EB	0.2733	0.1753	0.7423	−0.1716	−0.1048	0.4488	−0.1489
MSN	0.4367	−0.0573	−0.0211	−0.276	−0.6164	−0.2836	−0.2859
PNP	0.3682	0.3045	−0.1205	−0.4914	0.1658	−0.3902	0.4238
GWP	0.355	−0.3773	0.012	0.0982	0.356	−0.0706	−0.3124
GNP	0.45	0.0406	−0.0552	0.1846	0.5007	−0.0969	−0.3114
HGW	−0.1067	−0.5783	0.0516	0.0232	−0.2578	−0.3023	−0.1466
Yield	0.2919	−0.4196	0.3084	0.2806	−0.0137	−0.0272	0.6982
Eigenvalue	4.544427	2.763194	0.994832	0.401213	0.231626	0.064709	0
Contribution	0.5049	0.307	0.1105	0.0446	0.0257	0.0072	0
Cumulative contribution	0.5049	0.812	0.9225	0.9671	0.9928	1	1

Note: BPH, bottom pod height. PH, plant height. EB, number of effective branches. MSN, main stem nodes. PNP, pod number per plant. GWP, grain weight per plant. GNP, grain number per plant. HGW, hundred-grain weight.

**Table 2:** The scores and comprehensive rankings of different soybean germplasm accessions in PC1, PC2, and PC.

Germplasm	PC1	PC2	PC	Comprehensive Ranking
Hedou 29	3.872	0.0189	2.4151	1
He 20-3	−0.5454	2.8033	0.7208	2
Hedou12	1.4987	−1.1942	0.4804	3
Hedou 22	−0.7579	1.1894	−0.0216	4
He 23-75	−0.0531	−0.0204	−0.0407	5
He 22-85	−1.2669	−2.3903	−1.6917	6
He 19-97	−2.7474	−0.4068	−1.8624	7

**Table 3:** The scores and comprehensive rankings of different agronomic traits of different soybean germplasm accessions based on the membership function method.

Name	BPH	PH	EB	MSN	PNP	GWP	GNP	HGW	Yield	Sum	Ranking
Hedou 29	0.2118	1	0.625	1	1	1	1	0.2675	0.8173	6.9215	1
Hedou 12	0.6118	0.498	1	0.8333	0.3878	0.7485	0.4415	0.6776	1	6.1985	2
He 23-75	0.9529	0.5102	0.5	0.6667	0.7245	0.406	0.2554	0.4601	0.2623	4.7381	3
He 22-85	1	0.2735	0.25	0.3333	0.0306	0.6636	0.1877	1	0.7757	4.5144	4
Hedou 22	0.6235	0	0.875	0.1667	0.5	0.3265	0.36	0.1976	0.3308	3.3801	5
He 20-3	0	0.2	0.625	0.4444	0.6327	0	0.2662	0	0	2.1683	6
He 19-97	0.8118	0.4041	0	0	0	0.1965	0	0.5758	0.1212	2.1094	7

Note: BPH, bottom pod height. PH, plant height. EB, number of effective branches. MSN, main stem nodes. PNP, pod number per plant. GWP, grain weight per plant. GNP, grain number per plant. HGW, hundred-grain weight.

**Table 4:** Reference standard for salt tolerance level of soybean at germination stage.

Salt Tolerant Level	Relative Germination Rate Classification	Salt Tolerance Type
1	90% < Relative germination rate ≤ 100%	Extremely strong
2	70% < Relative germination rate ≤ 90%	Strong
3	50% < Relative germination rate ≤ 70%	Moderate
4	30% < Relative germination rate ≤ 50%	Sensitive
5	Relative germination rate ≤ 30%	Extremely sensitive

## Data Availability

Data are contained within the article and Supplementary Materials.

## Supplementary Materials

The following supporting information can be downloaded at https://www.mdpi.com/article/10.3390/plants15192991/s1, Table S1: The relative germination rate, salt tolerance level and salt tolerance type of experimental soybean germplasm accessions; Table S2: Names of experimental soybean germplasm accessions.

## Abbreviation

The following abbreviation is used in this manuscript:

PCA	Principal component analysis
