# Supplementary material for: Comprehensive Evaluation of the Salt Tolerance of Soybean Germplasm Resources Cultivated by Heze Academy of Agricultural Sciences
Source: Plants (Basel). 2026 Sep 30;15(19):2991. doi: 10.3390/plants15192991 (PMC13667129; doi:10.3390/plants15192991)
Supplement: Supplementary file 1 [file plants-15-02991-s001.zip › plants-4555741-supplementary.pdf]

Table S1. The relative germination rate, salt tolerance level and salt tolerance type of experimental soybean germplasm accessions.

Number	Name	Relative germination rate	Standard deviation	Salt tolerant level	Salt tolerance type
1	Hedou 12	86.54%	9.99%	2	Strong
2	Hedou 13	69.70%	5.25%	3	Medium
3	Hedou 14	57.89%	5.26%	3	Medium
4	Hedou 15	20.00%	8.66%	5	Extremely sensitive
5	Hedou 16	7.14%	12.37%	5	Extremely sensitive
6	Hedou 17	64.52%	5.59%	3	Medium
7	Hedou 18	18.18%	7.87%	5	Extremely sensitive
8	Hedou 19	9.09%	15.75%	5	Extremely sensitive
9	Hedou 20	12.50%	21.65%	5	Extremely sensitive
10	Hedou 21	30.95%	4.12%	4	Sensitive
11	Hedou 22	73.68%	9.12%	2	Strong
12	Hedou 23	0.00%	0.00%	5	Extremely sensitive
13	Hedou 28	46.15%	0.00%	4	Sensitive
14	Hedou 29	78.95%	0.00%	2	Strong
15	Hedou 32	29.63%	6.42%	5	Extremely sensitive
16	Hedou 33	28.57%	6.19%	5	Extremely sensitive
17	Hedou 36	30.77%	26.65%	4	Sensitive
18	Hedou 37	60.00%	6.67%	3	Medium
19	Hedou 38	35.00%	8.66%	4	Sensitive
20	Hedou 39	50.00%	5.41%	4	Sensitive
21	Hedou 41	46.43%	6.19%	4	Sensitive
22	Hedou 45	28.95%	4.56%	5	Extremely sensitive
23	He 19-37	26.32%	9.12%	5	Extremely sensitive
24	He 18-74	51.61%	5.59%	3	Medium
25	He 19-49	25.00%	6.19%	5	Extremely sensitive
26	He 18-87	29.41%	10.19%	5	Extremely sensitive
27	He 19-97	73.68%	9.12%	2	Strong
28	He 22-18	9.09%	7.87%	5	Extremely sensitive
29	He 9-18	45.95%	9.36%	4	Sensitive
30	He 19-51	15.91%	3.94%	5	Extremely sensitive
31	He 20-43-1	47.06%	5.09%	4	Sensitive
32	He 20-43-2	50.00%	5.41%	4	Sensitive
33	He 21-73	32.50%	4.33%	4	Sensitive
34	He 21-78	37.50%	0.00%	4	Sensitive
35	He 21-57	40.48%	4.12%	4	Sensitive
36	He 22-13	56.76%	8.11%	3	Medium
37	He 22-58	42.86%	10.71%	4	Sensitive
38	He 22-85	78.38%	9.36%	2	Strong

Number	Name	Relative germination rate	Standard deviation	Salt tolerant level	Salt tolerance type
39	He 22-74	48.65%	0.00%	4	Sensitive
40	He 22-111	58.62%	3.00%	3	Medium
41	He 18-84	33.33%	8.88%	4	Sensitive
42	He 19-29-2	65.12%	4.03%	3	Medium
43	He 20-108	66.67%	0.00%	3	Medium
44	He 20-118	50.00%	8.33%	4	Sensitive
45	He 21-40	63.41%	8.45%	3	Medium
46	He 21-86	29.41%	10.19%	5	Extremely sensitive
47	He 19-84	32.43%	8.11%	4	Sensitive
48	He 22-106	20.00%	0.00%	5	Extremely sensitive
49	He 17-109	50.00%	10.83%	4	Sensitive
50	He 22-64	40.00%	3.46%	4	Sensitive
51	He 22-82	43.59%	4.44%	4	Sensitive
52	He 23-75	79.31%	11.95%	2	Strong
53	He 23-82	37.93%	11.95%	4	Sensitive
54	He 23-143	47.06%	10.19%	4	Sensitive
55	He 22-54	62.00%	9.17%	3	Medium
56	He 22-121	52.17%	13.04%	3	Medium
57	He 20-31	27.03%	4.68%	5	Extremely sensitive
58	He 22-114	37.04%	6.42%	5	Extremely sensitive
59	He 19-1-1	38.24%	5.09%	4	Sensitive
60	He 20-3	73.47%	6.12%	2	Strong
61	He 18-9	54.29%	9.90%	3	Medium
62	He 19-2	38.46%	6.66%	4	Sensitive
63	He 14-59	69.23%	0.00%	3	Medium
64	He 22-102	47.06%	10.19%	4	Sensitive
65	He 19-52	47.06%	10.19%	4	Sensitive
66	He 20-106	38.46%	7.69%	4	Sensitive
67	He 18-28	63.41%	4.22%	3	Medium
68	He 22-62	55.00%	4.33%	3	Medium
69	He 22-119	68.00%	6.93%	3	Medium
70	He 22-127	50.00%	7.87%	4	Sensitive
71	He 19-55	34.37%	5.41%	4	Sensitive
72	He 14-30	63.64%	0.00%	3	Medium
73	He 20-1	52.38%	8.25%	3	Medium
74	He 16-25	42.86%	14.29%	4	Sensitive
75	He 1715	42.86%	10.71%	4	Sensitive
76	He 16-19	6.67%	11.55%	5	Extremely sensitive
77	He 19-30	50.00%	10.00%	4	Sensitive
78	He 1877	50.00%	10.83%	4	Sensitive
79	He 18-37	22.45%	3.53%	5	Extremely sensitive
80	He 20-63	44.00%	6.93%	4	Sensitive

Number	Name	Relative germination rate	Standard deviation	Salt tolerant level	Salt tolerance type
81	He 18-67-1	29.79%	3.69%	5	Extremely sensitive
82	He 19-80	37.50%	9.38%	4	Sensitive
83	He 19-101-1	67.44%	10.66%	3	Medium
84	He 21-48	28.21%	4.44%	5	Extremely sensitive
85	He 21-60	65.31%	3.53%	3	Medium
86	He 21-67	52.17%	13.04%	3	Medium
87	He 21-45	43.24%	4.68%	4	Sensitive
88	He 22-100	46.15%	0.00%	4	Sensitive
89	He 22-105	21.74%	7.53%	5	Extremely sensitive
90	He 23-42	29.27%	0.00%	5	Extremely sensitive
91	He 23-103	36.36%	9.09%	4	Sensitive
92	He 23-111	36.59%	0.00%	4	Sensitive
93	He 23-1	46.43%	6.19%	4	Sensitive
94	He 21-79	37.14%	4.95%	4	Sensitive
95	He 22-70	38.46%	6.66%	4	Sensitive
96	He 22-93	9.52%	8.25%	5	Extremely sensitive
97	He 22-96	9.52%	8.25%	5	Extremely sensitive
98	He 23-77	44.44%	11.11%	4	Sensitive
99	He 23-81	34.15%	4.22%	4	Sensitive
100	He 23-84	54.00%	10.39%	3	Medium
101	He 23-102	48.15%	12.83%	4	Sensitive
102	He 23-106	54.84%	5.59%	3	Medium
103	He 23-115	48.28%	11.95%	4	Sensitive
104	He 23-120	50.00%	12.37%	4	Sensitive
105	He 23-128	46.67%	5.77%	4	Sensitive
106	He 24-16	27.78%	4.81%	5	Extremely sensitive
107	He 24-28	57.58%	10.50%	3	Medium
108	He 24-35	53.85%	7.69%	3	Medium
109	He 24-67	26.92%	6.66%	5	Extremely sensitive
110	He 24-76	24.00%	12.00%	5	Extremely sensitive
111	He 24-90	46.88%	9.38%	4	Sensitive
112	He 24-96	45.45%	7.87%	4	Sensitive
113	He 24-98	53.70%	3.21%	3	Medium
114	He 24-103	38.10%	8.25%	4	Sensitive
115	He 24-104	23.08%	0.00%	5	Extremely sensitive
116	He 24-105	26.67%	11.55%	5	Extremely sensitive

Table S2. Names of experimental soybean germplasm accessions.

Number	Name
1	Hedou 12
2	Hedou 13
3	Hedou 14
4	Hedou 15
5	Hedou 16
6	Hedou 17
7	Hedou 18
8	Hedou 19
9	Hedou 20
10	Hedou 21
11	Hedou 22
12	Hedou 23
13	Hedou 28
14	Hedou 29
15	Hedou 32
16	Hedou 33
17	Hedou 36
18	Hedou 37
19	Hedou 38
20	Hedou 39
21	Hedou 41
22	Hedou 45
23	He 19-37
24	He 18-74
25	He 19-49
26	He 18-87
27	He 19-97
28	He 22-18
29	He 9-18
30	He 19-51
31	He 20-43-1
32	He 20-43-2
33	He 21-73
34	He 21-78
35	He 21-57
36	He 22-13
37	He 22-58
38	He 22-85

Number	Name
39	He 22-74
40	He 22-111
41	He 18-84
42	He 19-29-2
43	He 20-108
44	He 20-118
45	He 21-40
46	He 21-86
47	He 19-84
48	He 22-106
49	He 17-109
50	He 22-64
51	He 22-82
52	He 23-75
53	He 23-82
54	He 23-143
55	He 22-54
56	He 22-121
57	He 20-31
58	He 22-114
59	He 19-1-1
60	He 20-3
61	He 18-9
62	He 19-2
63	He 14-59
64	He 22-102
65	He 19-52
66	He 20-106
67	He 18-28
68	He 22-62
69	He 22-119
70	He 22-127
71	He 19-55
72	He 14-30
73	He 20-1
74	He 16-25
75	He 1715
76	He 16-19
77	He 19-30

Number	Name
78	He 1877
79	He 18-37
80	He 20-63
81	He 18-67-1
82	He 19-80
83	He19-101-1
84	He 21-48
85	He 21-60
86	He 21-67
87	He 21-45
88	He 22-100
89	He 22-105
90	He 23-42
91	He 23-103
92	He 23-111
93	He 23-1
94	He 21-79
95	He 22-70
96	He 22-93
97	He 22-96
98	He 23-77
99	He 23-81
100	He 23-84
101	He 23-102
102	He 23-106
103	He 23-115
104	He 23-120
105	He 23-128
106	He 24-16
107	He 24-28
108	He 24-35
109	He 24-67
110	He 24-76
111	He 24-90
112	He 24-96
113	He 24-98
114	He 24-103
115	He 24-104
116	He 24-105
